# The role and safety of UVA and UVB in UV-induced skin erythema

**DOI:** 10.3389/fmed.2023.1163697

**Published:** 2023-06-27

**Authors:** Jing-Wen Yang, Guo-Biao Fan, Fei Tan, Hai-Mei Kong, Qing Liu, Ying Zou, Yi-Mei Tan

**Affiliations:** ^1^Department of Skin and Cosmetics Research, Shanghai Skin Disease Hospital, School of Medicine, Tongji University, Shanghai, China; ^2^Professional Technical Service Platform for Clinical Evaluation of Skin Health Related Products, Shanghai Science and Technology Commission, Shanghai, China; ^3^MPA Key Laboratory for Monitoring and Evaluation of Cosmetics, Shanghai, China; ^4^Department China Regulatory Affairs, LVMH Perfume and Cosmetic (Shanghai) Limited Company, Shanghai, China; ^5^Human Phenome Institute, Fudan University, Shanghai, China

**Keywords:** UVA, UVB, minimal erythema dose, maximum blood flow depth, total blood flow, optical coherence tomography UVA, optical coherence tomography

## Abstract

**Background:**

Different wavelengths of ultraviolet (UV) light cause skin damage through different mechanisms. Minimal erythema dose (MED) is usually used to clinically evaluate skin sensitivity to ultraviolet radiation by inducing skin erythema using ultraviolet B (UVB) or ultraviolet A (UVA) + UVB.

**Aims:**

In this study, we detected changes in the blood flow at the MED erythema caused by UVB and UVA + UVB radiation through optical coherence tomography (OCT) to explain the role of different bands of ultraviolet rays in erythema induction.

**Methods:**

Two MED irradiation areas on the subjects' back were irradiated with UVB alone or UVA + UVB (UVA: UVB = 8:1). The absolute energy of UVB remained the same in UVB and UVA+UVB. At 24 h after the irradiation, the changes in the blood flow in the MED area were detected using OCT.

**Results:**

Compared with the blank control, the maximum blood flow depth, blood flow peak, and total blood flow of UVB-MED and UVA+UVB-MED were significantly increased. Notably, the maximum blood flow depth and blood flow peak of UVB-MED were higher than UVA+UVB-MED. There was no significant difference in total blood perfusion between UVA+UVB-MED and UVB-MED. Under the same UVB energy, the skin erythema caused by UVA + UVB was weaker than UVB alone.

**Conclusions:**

The analysis of local blood flow by OCT showed that the peak and maximum depth of local blood flow caused by UVB alone were significantly higher than UVA + UVB.

## Introduction

Ultraviolet is part of the electromagnetic spectrum with wavelengths between 100 and 400 nm, which is between visible light and x-rays (1, 2). According to the wavelength, ultraviolet is divided into ultraviolet A (UVA) (320–400 nm), ultraviolet B (UVB) (280–320 nm), and ultraviolet C (UVC) (100–280 nm) (3, 4). UV radiation is a mutagenic agent, and long-term overexposure to sunlight has been linked to photoaging and the development of skin cancer. Thus, it is considered one of the most common environmental factors that damage the skin's structure and function (5, 6). Different UV wavelengths cause skin damage through different mechanisms (7). UVA has a strong penetration ability into the skin through the cuticle, the epidermis, the dermis, and even subcutaneous tissue. The UVB penetrating ability of the skin is weak, which mainly causes damage to the epidermis and the superficial dermis (2, 8, 9). However, as the energy produced by ultraviolet decreases with increasing wavelength, UVB has a greater damaging effect on the epidermis than UVA (10, 11).

Erythema is the most apparent feature of ultraviolet radiation on the skin (12, 13, 47). It appears in the form of a visible redness of the skin that results from an increase in the blood volume of the superficial and deep dermal vessels for at least 6 h after UV exposure (12). This marked dilation of blood vessels can induce the formation of edema and the accumulation of white blood cells as an inflammatory response. At present, several studies have reported that ultraviolet irradiation, mainly UVB irradiation, can cause an increase in the local blood flow (12, 14–16, 46). Clinically, minimal erythema dose (MED) is usually used to evaluate skin sensitivity to ultraviolet radiation. MED refers to the minimum dose (J/m^2^) or the shortest time (s) in which erythema becomes visible on the skin 24 h after irradiation (17). While UVA is not primarily responsible for erythema, it does play a significant role in pigmentation (11, 18, 19). Practically, both UVA and UVB are used in tandem, not UVB alone, to induce skin erythema. Therefore, the role of UVA in erythema induction merits further discussion.

Optical coherence tomography (OCT) is a non-invasive diagnostic technique with an effective scanning depth of 2 mm (20). The technique is similar to ultrasound scanning, but OCT has a much higher resolution (< 10.0 um) at the same imaging depth because it is near-infrared (21). Dynamic OCT, also called speckle variance OCT or optical microangiography, is based on optical Doppler tomography, which combines Doppler velocimetry and optical coherence tomography to measure the movement velocity of blood cells at discrete spatial positions in skin tissue, and can capture microvascular blood flow with a blood flow rate as low as 20 mm/s (20, 22–24). Therefore, dynamic OCT enables the visualization and measurement of blood vessel morphology, providing a new perspective on skin health, inflammation, and tumor lesions. Dynamic OCT combines conventional OCT imaging with blood flow data to produce a living image of skin microvessels. Significantly, dynamic optical coherence tomography (OCT) has demonstrated its immense value in characterizing inflammatory processes and discerning the functional and metabolic attributes of various skin diseases (25). A number of studies have employed dynamic OCT to scrutinize the microvascular architecture and changes in blood flow within acne lesions. These studies indicate an augmented vascular signal proximal to comedones, as observed in the dynamic OCT en-face view (26). Intriguingly, the vascular signal in the apparently unaffected skin of acne patients was found to be not significantly different from that in the skin of healthy volunteers without acne (25).

The present study used UVB and UVA + UVB to irradiate skin to induce erythema and then detected the changes in the blood flow at the MED erythema caused by UVB and UVA + UVB radiation through OCT to explore the role of two different UV lights in causing skin erythema.

## Subjects and methods

### Subjects

A total of 124 subjects from Shanghai were recruited, including 55 men and 69 women aged 20–59 years, with an average age of 32.30 ± 6.06 years. The study was conducted in accordance with the Declaration of Helsinki, approved by the ethics committee of our institution, and registered at the Chinese Clinical Trial Registry. According to the Fitzpatrick classification method, the skin light classification of all the subjects was type III. We chose the back as the test site. Subjects were required to sign an informed consent form prior to participating in the trial. The exclusion criteria included (1) subjects who were pregnant or breast-feeding women or who were planning for pregnancy; (2) subjects who had eczema, psoriasis, and so on; (3) subjects who had participated in the same test within the last 3 months or had participated in the same test before 3 months but the melanic marks on the test site had not been completely removed; and (4) subjects who used products or drugs (e.g., hydroquinones) that could affect skin color in the last 3 months.

## Instruments and methods

### The solar simulator

The solar simulator used was the SOLAR LIGHT's Model 601–300 Multiport (Solar Light Co., Philadelphia, Pennsylvania). This multiport xenon arc solar simulator (300 W), equipped with Schott WG320 and UG 11/1mm filters, emits a continuous spectrum from 290 nm to 400 nm, similar to sunlight. UV radiation was conducted using six optic fibers (8 mm in diameter) defined on the skin. The radiation energy delivered by the solar simulator was measured using a PMA 2100 radiometer (Solar Light Company).

### OCT equipment

The test sites were scanned with OCT (VivoSight OCT Scanner, MDL) over an area of 6 ^*^ 6 mm, a depth of 0–2 mm, and an optical resolution of < 7.5 μm laterally and < 5 μm axially. The function setting automatically provided 60 lateral scans of 6 mm length every 100 μm. No preparation of the skin surface was required, and no oil or ointment was used with the device. The dynamic OCT images were evaluated using Vivo tools. This software tool was specifically designed for processing OCT image data acquired with the VivoSight OCT scanner. It enables the extraction of valuable quantitative microcirculation information, such as the depth of the blood flow, peak blood flow, and total blood flow perfusion at the test sites.

### Study procedures

**Figure 1:**
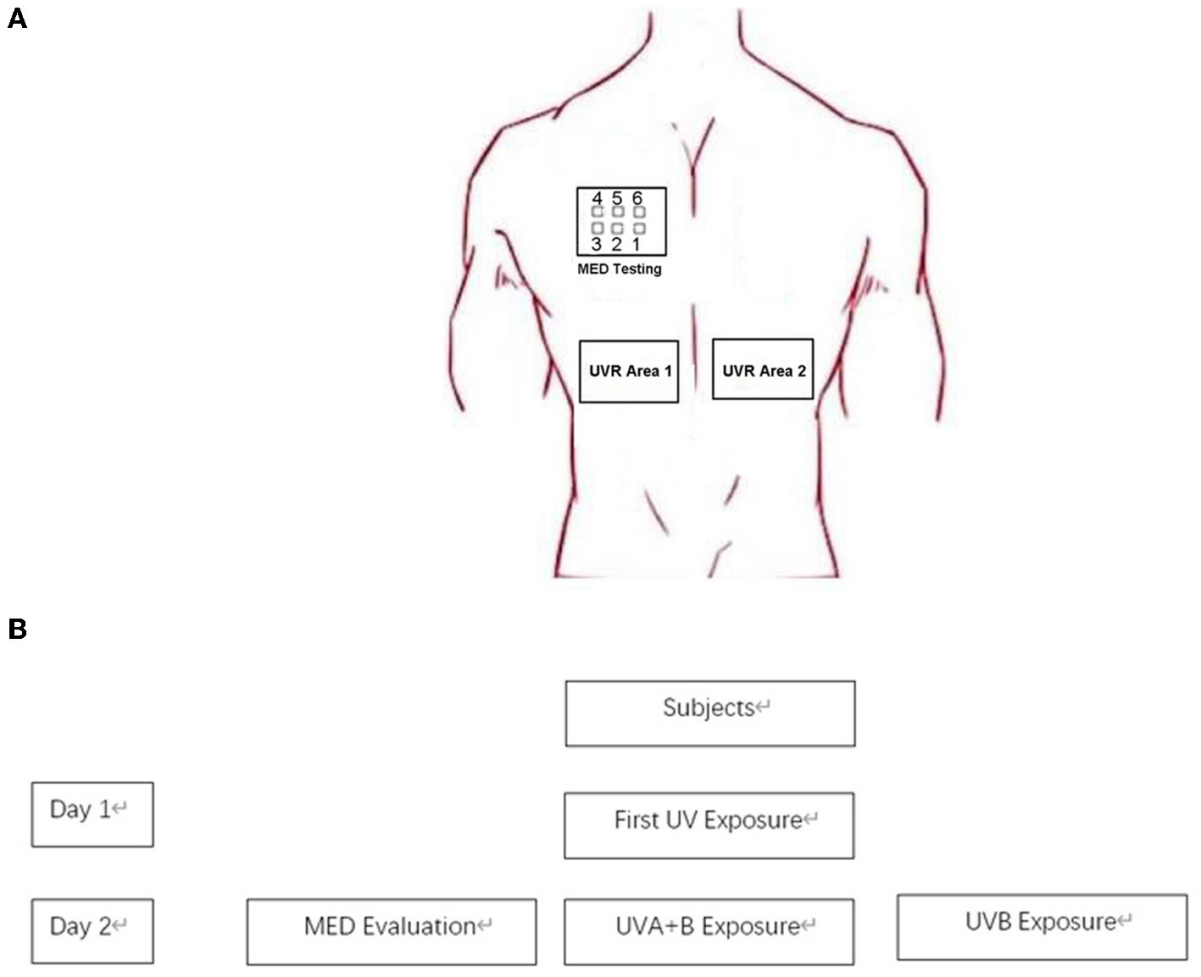
Simplified overview of the study process. **(A)** Test site on subjects. **(B)** Study process. On the first day, the initial UV exposure (UVA + B) was administered to the subjects' upper back. After a period of 24 h, minimal erythema dose (MED) was assessed. Subsequently, UVA + B and UVB were separately exposed on the subjects' backs, with the dosage of the MED set on the third port of six.

**Figure 2:**
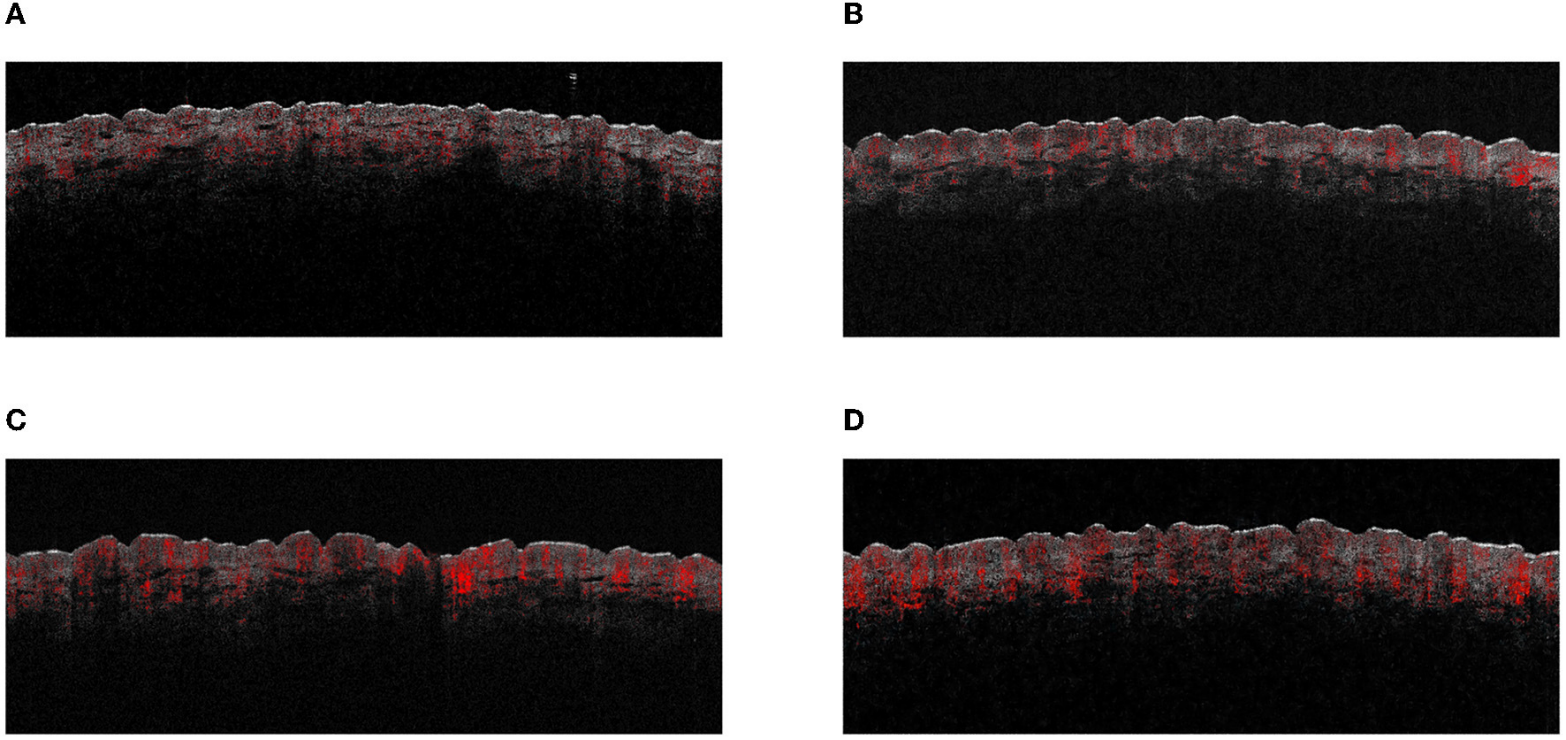
The OCT images of different test areas. **(A)** UVB control test site. **(B)** UVA+B control test site. **(C)** UVB-induced MED test site. **(D)** UVA+B-induced MED test site.

We chose the skin on both sides of the spine on the back as the test and control areas; the bra indentation area was avoided in women. The MED meter consisted of six illumination perforations with decreasing doses. Two minimal erythema dose (MED) irradiation areas were selected on the back and subjected to either UVB alone or a combination of UVA and UVB (UVA: UVB = 8:1), mirroring the same ratio present in sunlight during daylight hours. The absolute energy of UVB was maintained consistently between the UVB only and the combined UVA and UVB groups. All procedures, including MED inductions, were conducted in compliance with the methods outlined in ISO 24444 (2019) (27). At 24 h after the end of irradiation, the MED erythema area caused by UVA + UVB was used as the test area, and the change in the blood flow on the MED erythema area after UVB or UVA + UVB irradiation was detected using OCT. This detailed process is depicted in Figure 1. Importantly, the clinical visual scores for both UVB-MED and UVA+UVB-MED were recorded as “+” according to the patch test result recommendations by the International Contact Dermatitis Research Group (ICDRG) (28). The test sites that were not exposed to UV were set as controls. There were two control sites: one beside the UVB exposure site and another beside the UVA+B site. The OCT image of different test sites is shown in Figure 2.

### Statistics

Quantitative data are presented as means and standard deviations (SD). The Kolmogorov–Smirnov normality test was used for quantitative data to determine whether the data were parametric or non-parametric. The paired *t*-test was used for parametric data, and the Wilcoxon signed-rank test was used for non-parametric data. The significant level was set at 0.05. The analysis was performed using SPSS 22.0 for Windows (IBM, USA).

## Results

### The maximum blood flow depth in UVB and UVA+UVB-irradiated areas

Compared with the blank control, the maximum blood flow depth of UVB-MED and UVA+UVB-MED was significantly increased (*P* < 0.01). There was no significant difference in maximum blood flow depth at the blank control in the two MED measurement areas. Compared with the maximum blood flow depth of UVA+UVB-MED, the maximum blood flow depth of UVB-MED was significantly increased (*P* < 0.05, Table 1).

**Table 1:** Maximum blood flow depth in the UVB and UVA+UVB-irradiated areas (mm).

**UVB**		**UVA** + **UVB**	
**Blank control**	**UVB-MED**	**Blank control**	**UVA** + **UVB-MED**
0.93 ± 0.30	1.08 ± 0.26^***#^	0.91 ± 0.27	1.00 ± 0.26^**^

^**^Compared with the blank control *P* < 0.01, ^***^P < 0.001; ^#^Compared with UVA+UVB-MED, *P* < 0.05.

### The blood flow peak in UVB and UVA+UVB-irradiated areas

Compared with the blank control, the blood flow peak of UVB-MED and UVA+UVB-MED was significantly increased (*P* < 0.001). There was no significant difference in blood flow peak at blank control in the two MED measurement areas. Compared with the blood flow peak of UVA+UVB-MED, the blood flow peak of UVB-MED was significantly higher (*P* < 0.05, Table 2).

**Table 2:** Blood flow peak in UVB and UVA+UVB-irradiated areas (OD).

**UVB**		**UVA** + **UVB**	
**Blank control**	**UVB-MED**	**Blank control**	**UVA** **+** **UVB-MED**
0.10 ± 0.02	0.11 ± 0.02^***#^	0.10 ± 0.02	0.11 ± 0.01^***^

^***^Compared with the blank control *P* < 0.001, ^#^Compared with UVA+UVB-MED, *P* < 0.05.

### Total blood perfusion in UVB and UVA+UVB-irradiated areas

Compared with the blank control, the total blood perfusion of UVB-MED and UVA+UVB-MED significantly increased (*P* < 0.01). There was no significant difference in skin total blood perfusion at blank control in the two MED measurement areas. There was also no significant difference in skin total blood perfusion between UVA+UVB-MED and UVB-MED (Table 3).

**Table 3:** Total blood perfusion in UVB and UVA+UVB-irradiated areas (a.u.).

**UVB**		**UVA** + **UVB**	
**Blank control**	**UVB-MED**	**Blank control**	**UVA** **+** **UVB-MED**
0.63 ± 0.20	0.73 ± 0.18^***^	0.65 ± 0.18	0.69 ± 0.15^**^

^**^Compared with the blank control *P* < 0.01, ^***^ *P* < 0.001.

## Discussion

While UV radiation is crucial for life, excessive exposure, particularly to UVB, can precipitate various health concerns, including DNA and protein structural damage and premature skin aging (29). Acute damage from UVB irradiation can present as sunburn, photoaging, and melanoma, each of which carries significant health risks. Due to UVB's high intensity and short wavelength, it causes considerable damage to the epidermis (30). UVB is also categorized as carcinogenic because of its potential to induce skin cancer after prolonged exposure (31). Erythema represents the skin's initial inflammatory response to UV irradiation.

**Figure 3:**
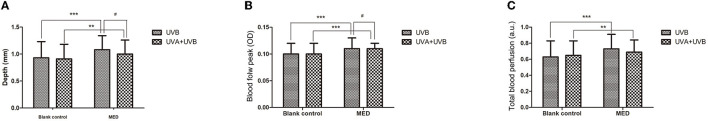
Results of dynamic parameters analyzed by OCT. **(A)** Maximum depth of blood flow. **(B)** Blood flow peak values. **(C)** Total blood perfusion. **Compared with the blank control *P* < 0.01, ***P < 0.001; ^#^Compared with UVA+UVB-MED, *P* < 0.05.

Consequently, in clinical diagnosis and research, ultraviolet lights are usually used to induce erythema, and MED is used to carry out studies and investigate skin-related diseases. Justus et al. proved that, in healthy subjects whose forearm skin was irradiated with ultraviolet light with a wavelength of 270–380 mn in a 3 cm-diameter area, the blood flow increased in a dose-dependent manner and peaked 12 and 36 h after irradiation, respectively (12). Other studies have also confirmed that acute UV exposure causes an increase in blood flow to the skin (14, 32, 33). UVA primarily induces immediate pigment darkening (IPD) and persistent pigment darkening (PPD), while UVB predominantly causes delayed tanning (DT), which is often preceded by erythema. Therefore, UVB is more erythemogenic than UVA (11, 15, 18, 19). Although erythema is typically triggered by UVB, a combined UVA and UVB radiation source is usually employed in practice to induce the erythema irritation model. As such, the role of UVA in the process of erythema induction is certainly worth discussing. Considering the above reasons, we did not set UVA irradiation alone in this study. In this study, skin erythema was induced using UVA + UVB or UVB alone, and the differences in the erythema induced by UVA + UVB and UVB were compared under the same UVB energy. The results showed that skin erythema caused by UVA + UVB was weaker than those caused by UVB alone. The analysis of local blood flow by OCT showed that the peak and maximum depth of local blood flow caused by UVB alone were significantly higher than those caused by UVA+UVB. The analyzed results are shown in Figure 3.

As a non-invasive optical technology, OCT is widely used to detect changes in skin blood flow, including skin damage caused by ultraviolet light. The blood flow measured by OCT depends on the number of red blood cells in the field of vision and the average speed of red blood cells (34). As an established and mature microvascular assessment method, OCT enables the rapid, non-invasive analysis of blood flow patterns in the skin, thus providing continuous and accurate measurements (34–36). Current studies have also demonstrated that OCT is an effective method for detecting, locating, and quantifying changes in skin blood flow, especially for assessing changes in microvascular blood flow (37). Ou Qin et al. utilized OCT to evaluate changes in blood flow following treatments with sodium lauryl sulfate and tape stripping on the skin. They observed a notable increase in blood flow volume in the sodium lauryl sulfate group 1 day after the intervention compared to the distilled water group. This elevated blood flow volume subsequently declined gradually, reverting to the levels observed in the control group by the third-day post-intervention.

Conversely, tape stripping did not impact blood flow volume from Day 1 to Day 3 (38). OCT is sensitive to the movement of blood cells, thus providing complete information about the shape of blood vessels and blood flow data that reaches the maximum depth of the skin in the middle dermis (21, 39, 45). OCT has a good correlation and repeatability in assessing changes in skin blood flow (40, 41) and has the advantages of locating the dermal-epidermal junction and quantifying the size and distribution of blood vessels (40). In this study, OCT was used to detect the blood flow distribution in the MED erythema area, which measured the maximum subcutaneous depth of blood flow and calculated the total and the peak of blood flow in the test areas. These parameters can objectively evaluate the severity of skin erythema.

## Conclusion

Although UVB is the main ultraviolet band causing skin erythema, UVA + UVB is usually used to induce MED. Our study found that the intensity of erythema induced by UVA + UVB was weaker than that induced by UVB. Under the same total amount of local subcutaneous blood perfusion, UVA + UVB could reduce the peak value of local blood flow and cause a shallow abnormal maximum depth of subcutaneous blood flow. Therefore, UVA + UVB is safer in reducing the degree of skin injury than UVB. We believe that UVA has strong penetration and can reach the dermis, thereby activating macrophages and plasma cells in the subcutaneous tissue to participate in the regulation of immune response; UVA simultaneously promotes the expression of matrix metalloproteinase on dermis fibro-cells, reducing inflammatory cell infiltration and inflammatory response (42). Several studies have reported that UVA exposure can confer a degree of photoimmune protection against immunosuppression, with the photoimmune effect being contingent on the UVA dosage (37, 43). Reeve and his colleagues proposed that UVA radiation is not only immunologically benign but also generates photoproducts capable of counteracting radiation-induced immunosuppression. They suggested that, at least in mice, UVA radiation might exert a photoprotective effect if administered prior to UVB exposure (43). Furthermore, UVA radiation has been demonstrated to trigger an increase in the expression of heme oxygenase (HO), a surge that persisted for at least 3 days post-UVA exposure. This induction of HO can forestall UVB-induced immunosuppression by curbing oxidative stress (44). Our findings lend additional support to the concept of photoimmune protection against UVB. Therefore, when using ultraviolet light to induce skin erythema, using UVA + UVB as the light source can reduce skin inflammation and local blood flow peak and avoid strong skin erythema reactions, thus reducing skin damage.

## Data availability statement

The original contributions presented in the study are included in the article/supplementary material, further inquiries can be directed to the corresponding authors.

## Ethics statement

The studies involving human participants were reviewed and approved by the Ethics Committee of Skin Disease Hospital of Tongji University. The patients/participants provided their written informed consent to participate in this study.

## Author contributions

G-BF and J-WY designed the experiment and wrote the manuscript. FT, H-MK, and QL are the main members of the experiment, they conducted the experiment, and analyzed the data. G-BF, YZ, and Y-MT designed the experiment and edited the manuscript. All authors contributed to the article and approved the submitted version.
